# Artificial intelligence for livestock: a narrative review of the applications of computer vision systems and large language models for animal farming

**DOI:** 10.1093/af/vfae048

**Published:** 2025-01-04

**Authors:** Guilherme L Menezes, Gustavo Mazon, Rafael E P Ferreira, Victor E Cabrera, Joao R R Dorea

**Affiliations:** Department of Animal and Dairy Sciences, University of Wisconsin-Madison, Madison, WI 53703, USA; Department of Animal and Dairy Sciences, University of Wisconsin-Madison, Madison, WI 53703, USA; Department of Animal and Dairy Sciences, University of Wisconsin-Madison, Madison, WI 53703, USA; Department of Animal and Dairy Sciences, University of Wisconsin-Madison, Madison, WI 53703, USA; Department of Animal and Dairy Sciences, University of Wisconsin-Madison, Madison, WI 53703, USA; Department of Biological Systems Engineering, University of Wisconsin-Madison, Madison, WI 53703, USA

**Keywords:** machine learning, LLMs, natural language processing, precision farming, data-driven decision making

**Implications:**

- AI is already integrated into many daily tasks, including facial recognition and airport security, and its use is expanding into agriculture.
- AI supports data-driven decision-making in livestock production, particularly in dairy farming.
- Computer vision systems (CVS) are applied in dairy farming for animal identification, behavior monitoring, feeding intake, body weight estimation, and disease detection.
- Natural language processing (NLP) and large language models (LLMs) offer potential for optimizing data integration, dimensionality reduction, and knowledge retrieval in agriculture.
- There is a significant knowledge gap in the use of LLMs in animal farming, presenting opportunities for new research and technological advancements.

## Introduction

Artificial intelligence (AI) has become essential for decision-making across various industries. The number of research projects involving AI increased by 2.5 times between 2010 and 2018 compared to the previous decade (Lu, 2019). Companies investing in AI have seen significant improvements in sales, employment, and market value (Babina et al., 2024). This growth explains the increase in AI-related jobs from 2007 to 2018. By 2025, global AI investments are expected to reach 200 billion U.S. dollars (Goldman Sachs, 2023).

In agriculture and livestock, Babina et al. (2024) indicated a doubling of positions related to AI between 2015 and 2018 compared to the preceding seven years in the United States. Government initiatives, such as the 2021 investment by USDA-NIFA and NSF of $220 million in 11 new NSF-led AI Research Institutes, support the growth of precision livestock farming (USDA-NIFA, 2021). These investments facilitate sensor-based management, enabling large-scale animal phenotyping beyond traditional methods. Technologies developed through these initiatives can improve automation and decision-making in livestock systems, covering areas such as animal health, management, nutrition, and welfare.

There is a great variety of scientifically proven digital technologies currently available and under development for dairy cattle management. Among the technologies, the use of 2-dimensional (2D), 3-dimensional (3D), and thermal cameras combined with computer vision systems (CVS) offers researchers and producers a versatile, less laborious, and contact-free option for high-throughput phenotyping in livestock production (Gunaratnam et al., 2024). There is a plethora of CVS applications for dairy farming, ranging from animal identification to forage yield estimation in pasture systems. However, in this review, we focus on CVS applications that have been more extensively studied in the literature, which include animal identification, body weight (BW) and composition estimation, feed intake and behavior measurement, and lameness detection.

Additionally, this review includes a discussion on the use of transformer-based large language models (LLMs) in natural language processing (NLP) and multimodal AI applications and their ability to extract information from text, converting it into contextual embeddings for knowledge retrieval, text feature extraction, text classification, and text summarization (Min et al., 2023). LLMs can facilitate the democratization of knowledge due to their ability to synthesize vast amounts of information, such as the thousands of research papers published in the dairy science field annually (Kononoff, 2024).

Building on the increasing importance of AI in agriculture and livestock, this narrative review highlights key AI applications for dairy farming, with a specific focus on current and future applications of CVS and LLMs. This review explores how CVS models enhance decision-making, starting with animal identification and extending to farm-scale efficiency in areas such as BW, health, and welfare. Lastly, the section on LLMs summarizes key concepts about this novel AI application and discusses their current and potential applications in livestock farming.

## Computer Vision Systems

In this section, we discuss the use of CVS for applications related to animal identification, individual feed intake, behavior, health, and welfare. This study explored CVS because of its capacity to capture multiple phenotypes using a single camera. Therefore, studies focusing on 2D and 3D cameras utilizing different sensors, such as red, green, blue (RGB), and infrared, are highlighted.

### CVS for animal identification

Animal identification allows data to be collected on an individual level and is a crucial step for precision livestock farming. Traditional identification methods, such as ear tags and radio-frequency identification (RFID) systems, are labor-intensive, error-prone, and can impact animal welfare due to ear lesions (Awad, 2016). In contrast, CVS technologies do not require physical contact and have advanced significantly. In CVS, animal identification can be based on biometric features such as muzzle prints, retinal vascular patterns, and coat color (Allen et al., 2008; Li et al., 2017). Despite their high identification accuracy, this review will not further discuss the use of retinal vascular patterns and muzzle prints for animal identification, as their limited range of distance recognition and noise susceptibility might affect their successful adoption in commercial settings (Shojaeipour et al., 2021).

Animal identification using distinct coat color patterns, especially in Holstein cows, has been implemented in settings similar to commercial farms (Xiao et al., 2022) with a mean accuracy of over 89% (Zin et al., 2018; Bello et al., 2020; Xiao et al., 2022). Recently, Bergman et al. (2024) developed a system to identify dairy cows based on facial color patterns, achieving an F1 score of 0.928 using 1,230 face images of 77 cows. However, many dairy cattle breeds do not display distinct coat patterns, which makes identifying solid-colored animals challenging for CVS. Thus, researchers proposed the use of body shape for individual identification. For example, Ferreira et al. (2022) achieved an F1 score of 0.80 when combining depth images from the dorsal area and convolutional neural networks (CNNs) to identify individual calves among 38 animals.

**Figure 1.**
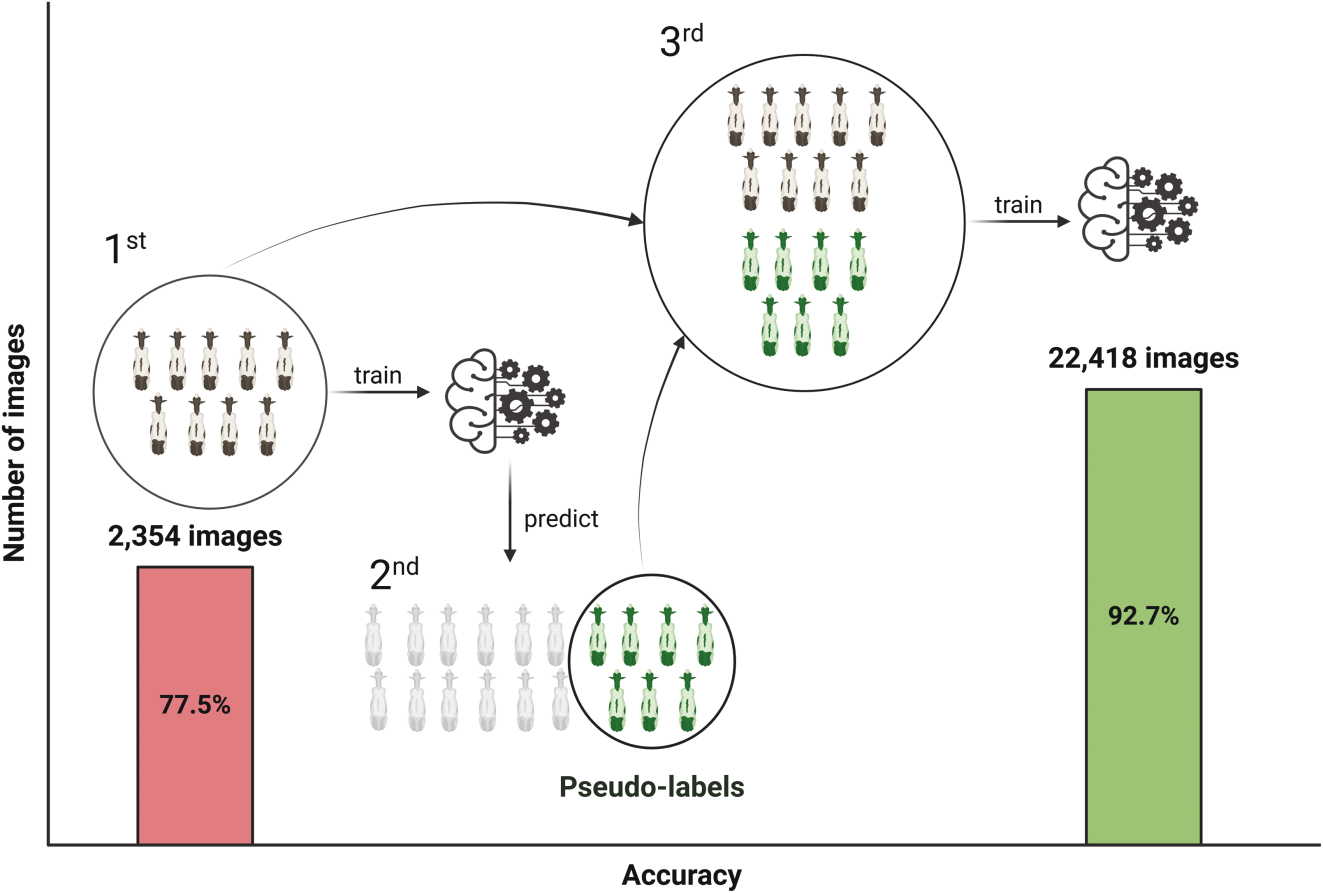
Graphical representation of the pseudo-labeling technique for individual identification utilized by (Ferreira et al., 2023). 1st represents the initial step of training with the labeled dataset. 2nd represents the incorporation of pseudo-labels that exceed a given threshold into a new training round. 3rd represents the model retrained with both the labeled data and the pseudo-labels. The bars represent the accuracy of the individual identification model before (bar on the left) and after (bar on the right) pseudo-labeling was adopted.

Despite achieving high accuracies, the use of CVS for animal identification requires large datasets (Xiao et al., 2022) to accurately identify an animal despite changes in appearance, behavior, or lighting conditions. In fact, larger datasets improve a model’s ability to generalize across factors such as lighting conditions, animal posture, occlusion, and camera angles (Das et al., 2024). The number of images needed to train satisfactory models can vary depending on herd size and environmental conditions. Although image labeling for training an AI model is done only once before on-farm implementation, it is labor-intensive and requires specialized labor. These specialized labor needs can limit the implementation of CVS in commercial settings. To address this issue, Ferreira et al. (2023) proposed the use of pseudo-labeling as a semisupervised learning technique. In this method, images that remain unlabeled after prediction are included in the training set, increasing the diversity of examples. This approach led to an observed accuracy increase from 77.5% to 92.7%. To demonstrate the process, a graphical representation of the technique adopted by Ferreira et al. (2023) is presented in Figure 1.

Improving the efficiency of animal identification using CVS requires addressing the challenge of identifying animals with open-set scenarios. Typically, research has concentrated on closed-set scenarios, where test animals have been previously seen in training data from different days. Therefore, the use of closed-set datasets to train CVS can limit their ability to recognize new animals. In contrast, models designed for open-set scenarios are more adaptable and capable of identifying new animals in the herd, being more applicable in real-life scenarios.

Andrew et al. (2021) applied the concept of open-set scenarios identification to a dataset of 46 Holstein cows using a Siamese Neural Network (SNN) to identify similarities between animal pairs and create embeddings classified by *k*-nearest neighbors. The model trained with 50% of unknown animals achieved 93.8% accuracy in individual identification. This method distinguishes unknown animals from known ones based on cluster distances, enabling alerts when a new animal is detected in the herd and providing valuable information for daily farm management. Applying this concept to solid-colored herds (Sharma et al., 2024) trained deep learning networks using depth images and PointNet, which converted these depth images into point clouds for animal identification based on body shape. The authors utilized combinations of softmax and reciprocal or regular triplet loss, similar to Andrew et al. (2021), and achieved an accuracy of 97%. Although these applications have the potential to be applied to any cattle breed, the authors emphasized that they need to be evaluated in real-world scenarios.

Applying animal identification at commercial farms is a current challenge and needs to be addressed in future studies. Although promising results have been reported, a significant knowledge gap remains in long-term animal tracking involving both coat color and 3D representations. Ferreira et al. (2022) achieved an F1 score of 0.86 using short-term tracking over three weeks by integrating depth images from the dorsal area with CNNs to identify individual calves among 5 animals. However, to our knowledge, no studies have evaluated animal identification over long periods. In real-world scenarios, open-set scenarios and long-term evaluations are crucial; typically, animals are identified early in life and tracked over the years at different farm locations depending on their life stage and production. Additionally, challenges such as occlusions, blurring, and varying lighting conditions during both day and night in farm settings also need to be addressed in future animal identification studies.

In addition to animal identification, CVS may also be used for other purposes, such as nutritional management and feed bunk evaluation. Thus, the next section of this review focuses on CVS approaches to improve nutritional (Bezen et al., 2020; Saar et al., 2022; Wang et al., 2023) and behavioral monitoring (Wu et al., 2021a; Bresolin et al., 2023) on-farm.

### Individual feed intake

Monitoring feed intake is fundamental for the nutritional management of dairy cows and calves. Furthermore, the monitoring of individual feed intake can provide valuable insights regarding animal nutrition, management, and health. Thus, in this section, we explore recent studies that utilized CVS to measure individual feed intake in dairy cattle.

Monitoring individual feed intake is laborious and requires specialized equipment, such as automated feed intake recording systems, which are mostly utilized for research purposes. However, current research utilizing CVS has demonstrated promising results for the future of on-farm individual intake estimation. For example, Bezen et al. (2020) proposed using RGB and depth sensors placed above the feed bunk and CNNs to predict meal size in lactating dairy cows. The authors reported a mean absolute error (MAE) of 0.241 and 0.127 kg/meal when using RGB or RGB and depth sensors, respectively. Similarly, Saar et al. (2022) used RGB and depth sensors placed above the feed bunk combined with CNNs to predict meal size in Holstein cows, achieving an MAE of 0.14 kg/meal. Recently, Wang et al. (2023) utilized RGB and depth sensors placed above feed bins combined with an SNN to predict meal size in dairy cows and reported an MAE of 0.10 kg/meal.

**Figure 2.**
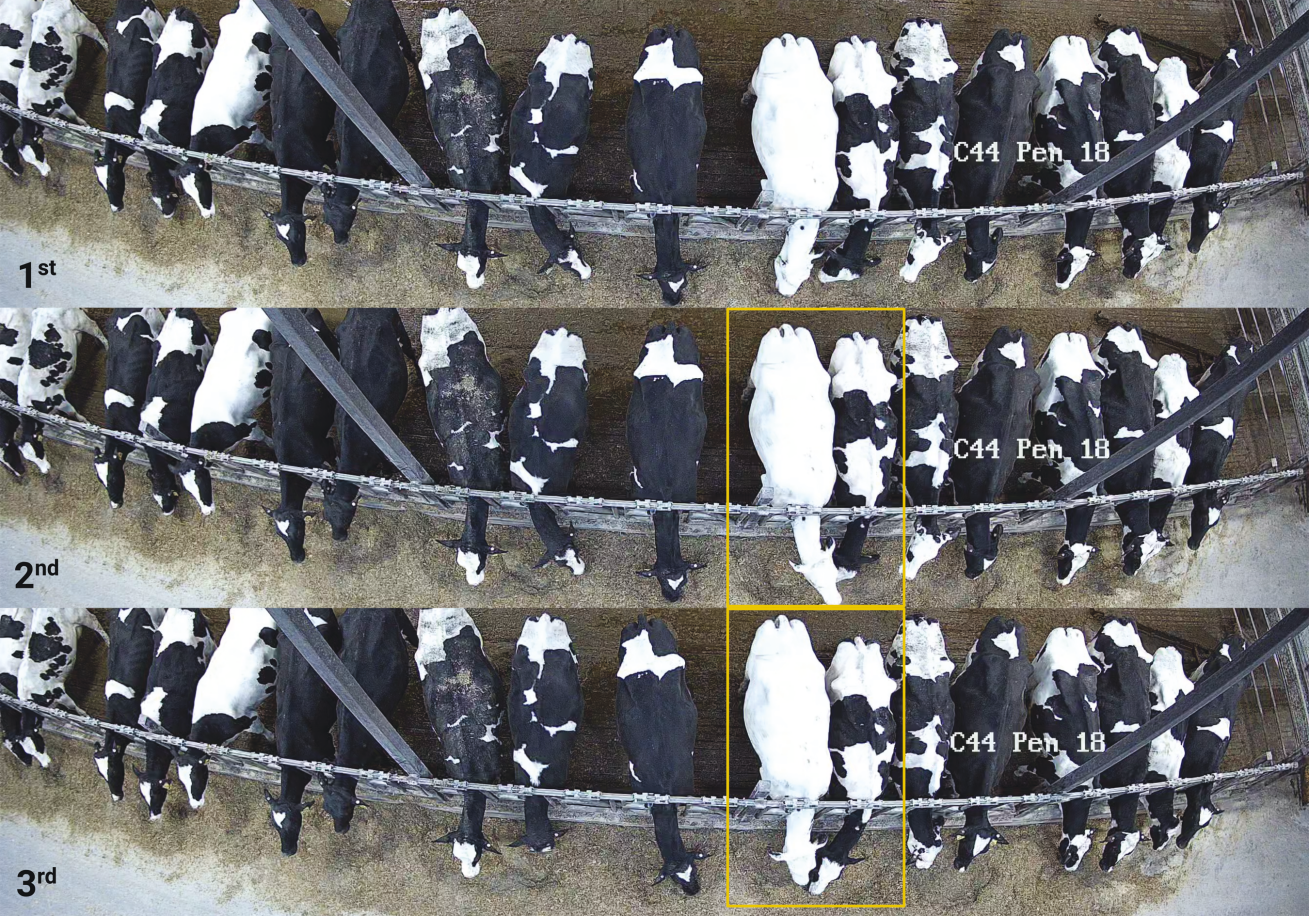
Examples of challenges that can be encountered during real-time feed intake estimation under commercial settings. 1st represents cows eating at the feed bunk without occlusion; 2nd represents one animal with complete head occlusion; and 3rd represents two cows eating from the same feed pile location.

Although promising results regarding feed intake estimation were reported in this section, the authors noted that the accuracy of their feed intake prediction models was impacted by lighting conditions. Hence, future studies should consider using other cameras, such as infrared night-vision cameras, in farm settings. Additionally, predicting individual feed intake by combining animal identification and feed pile disappearance estimation is challenging. As demonstrated in Figure 2, in farm settings such as free-stall, animals eat very close to each other, and particle disappearance might be due to feed particles being moved spatially but not necessarily ingested. Another potential issue is occlusions; typically, animals eating in the same place are often observed. Although these scenarios pose a challenge for individual feed intake estimation, they present an opportunity to measure animal behavior at the feed bunk. Thus, in the next section, we explore the literature on the use of CVS to measure animal behavior, a practical way to gain insights into the health, welfare, and performance of animals (Stygar et al., 2021).

### Animal behavior

The applications of monitoring animal behavior on farms are vast and can have practical applications associated with animal nutrition, reproduction, and health. In addition to monitoring individual feed intake, CVS can also monitor animal behaviors such as eating, drinking, ruminating, and resting. For example, Wu et al. (2021a) using RGB cameras combined with CNN and long short-term memory (LSTM) models, reported an accuracy of 97% when classifying drinking, ruminating, walking, standing, and lying behaviors of Holstein cows in outdoor areas. Furthermore, Bresolin et al. (2023) demonstrated the ability of CVS combined with an object detection algorithm to assess individual feeding behavior of group-housed dairy heifers. Their approach yielded *R*^2^ values of 0.39, 0.78, 0.63, and 0.99 for the number of visits, mean visit duration, interval between visits, and feeding time, respectively.

Although there are several applications for monitoring animal behavior using CVS, individual tracking reintroduces the challenge of accurate identification and re-identification in both closed and open-set scenarios. Additionally, depending on the camera position, such as a side view, occlusions of animals by bars and other animals are often observed. Combining all cameras to cover the entire barn is challenging due to the different views. For example, Lodkaew et al. (2023) utilized LightGBM to classify estrus behavior reporting an F1 score of 0.83. However, the authors reported that the F1 score decreased to 0.66 when individual identification algorithms were added to their model.

While identification remains the main bottleneck for individual behavior tracking, monitoring behaviors such as standing, lying, eating, and drinking shows promise in terms of the predictive quality of the models. In this context, there is an opportunity to utilize CVS for monitoring behaviors at a group level, making large-scale implementation more feasible. Thus, the development of more robust pipelines that are not negatively affected by environmental conditions or image occlusion is still needed. It is also important to highlight that animal growth and performance is associated with feed intake and behavior. Therefore, the next section reviews some of the latest publications utilizing CVS to predict animal growth and performance. These are important metrics for enhancing feed efficiency, resource utilization, and ultimately, sustainability in dairy farms.

### Animal growth and performance

Animal performance is a critical indicator driving management decisions on-farm. Hence, this section explores the application of CVS in predicting BW, growth, and body condition score (BCS). We emphasize these metrics due to their substantial impact on dairy farm operations. Milk production is a crucial component of dairy farm profitability, while measures related to growth, such as BW and hip width, are essential for evaluating replacement heifers. Additionally, body condition score (BCS) can serve as a valuable metric for animal nutritional status assessment and adjustment of management practices, especially around the transition period.

Measuring BW is a fundamental practice in evaluating the nutritional program and an animal’s energy status throughout its productive cycle. Besides the importance of evaluating BW, structural growth is also a critical indicator for assessing heifer development (Costa et al., 2021). In this context, Song et al. (2018) proposed the use of depth cameras to predict BW in Holstein cows using features such as hip width, days in milk (DIM), and parity, and reported a root mean square error of prediction (RMSEP) of 41 kg, representing a 5.2% error relative to the actual mean BWs. Recently, Gebreyesus et al. (2023) applied image processing to extract geometric features of different contours of the animal’s body to predict BW in Holstein and Jersey cows. The authors used CatBoost to predict BW, and their model achieved a Pearson correlation of 0.94 and an RMSEP of 33 kg, representing 4.0% of the observed values.

Continuous CVS monitoring can facilitate the extraction of features to fit growth curves and understand how body biometrics adjust to these curves. Evaluating skeletal measurements, such as height, hip width, and length, is equally or more important than BW because these measurements are not influenced by fat deposition and accurately reflect growth (Heinrichs et al., 1992). This information is crucial especially for first lactation cows, as those animals that are underperforming during the growth stage will partition energy to prioritize growth instead of milk yield (Van Amburgh et al., 2019). Thus, monitoring daily growth rates may provide farms with a valuable tool to adjust nutritional and breeding plans, avoid extra costs associated with overfeeding, and prevent lower growth rates that can influence age at first parturition and lactation performance.

Another promising application for CVS, especially for lactating cows, is the assessment of BCS. Measuring BCS is time-consuming and subjective, and small changes in body composition can be difficult to notice through visual inspection. Both 2D and 3D cameras can be used to assess BCS using CVS. The current literature on CVS to assess BCS in dairy cattle is extensive. Hence, in this section, we cover a few examples utilizing 2D and 3D cameras. For example, Wu et al. (2021b) used 2D cameras to compare the performance of CNNs and Vision transformers (ViT) in assessing BCS in dairy cows. The CNNs achieved an accuracy of 96.8% in estimating BCS with a 0.25 unit of difference, whereas ViT achieved an accuracy of 97.8%. Using 3D images to estimate shape and predict BCS has become more common and accurate. Utilizing 3D cameras placed at the exit of the milking parlor, Zhao et al. (2023) trained CNNs on feature images generated by calculating the vertical distances between each point in a voxel-based 3D point cloud and the convex hull that surrounds the cow’s rump area. The authors reported accuracies of 91% and 96% within a 0.25 and 0.50 unit difference from human estimation, respectively.

Although CVS using both 2D and 3D images shows high predictive accuracy, it is important to highlight that the validation of all these models was done by comparison with human visual assessments, which is subjective and error-prone. In addition, studies reported limited inclusions of cows with low or elevated BCS to validate their models. In fact, Mullins et al. (2019) validated a commercially available CVS for BCS in commercial settings and reported that the system was not equivalent to visual assessments when evaluating BCS in cows with BCS < 3.0 or BCS > 3.75. However, these systems measure BCS utilizing a continuous scale, which is different from the categorical system commonly used for visual BCS assessments. Stephansen et al. (2023) also assessed BCS prediction in 808 Jersey cows using 3D images. Their model achieved 48.1% accuracy for exact BCS predictions and 93.5% within a 0.5-unit deviation using a 70:30 train-test split. When evaluated on an external herd, accuracy ranged from 38.3% to 49.5% for exact BCS and 87.4% to 93.0% within a 0.5-unit deviation.

Truman et al. (2022) described the variations in automated BCS measurements throughout lactation and suggested the future use of automated BCS as a predictor for lactation performance and health status. Future studies should concentrate on using more quantitative standards, such as measures of contour, area, and shape of different parts of the body. Such initiatives would benefit farms by using these metrics to evaluate the risk of diseases related to negative energy balance, especially during the transition period. Although BW and BCS are utilized for on-farm decision-making, we must acknowledge that they can be associated with the animal’s health and welfare. Thus, in the next section of this review, we discuss a few of the latest studies utilizing CVS to monitor dairy cow health and welfare.

### Health and welfare

Maintaining healthy and stress-free cows is one of the most important and challenging goals on dairy farms, especially considering all the negative economic and animal welfare impacts associated with diseases and stressors. Lameness and mastitis are two of the most prevalent and costly disorders that directly affect dairy cow performance, health, and welfare. Similarly, decreases in production, poor health, and welfare associated with heat stress can be observed in cows across the globe (Becker et al., 2020). Furthermore, the management of lameness, mastitis, and heat stress relies mostly on animal monitoring and prevention strategies. Thus, there is an opportunity to utilize CVS systems to detect and predict these disorders. In this section, this study explores the current literature utilizing CVS to monitor dairy cow health and welfare, with a focus on detecting lameness, mastitis, and heat stress.

**Figure 3.**
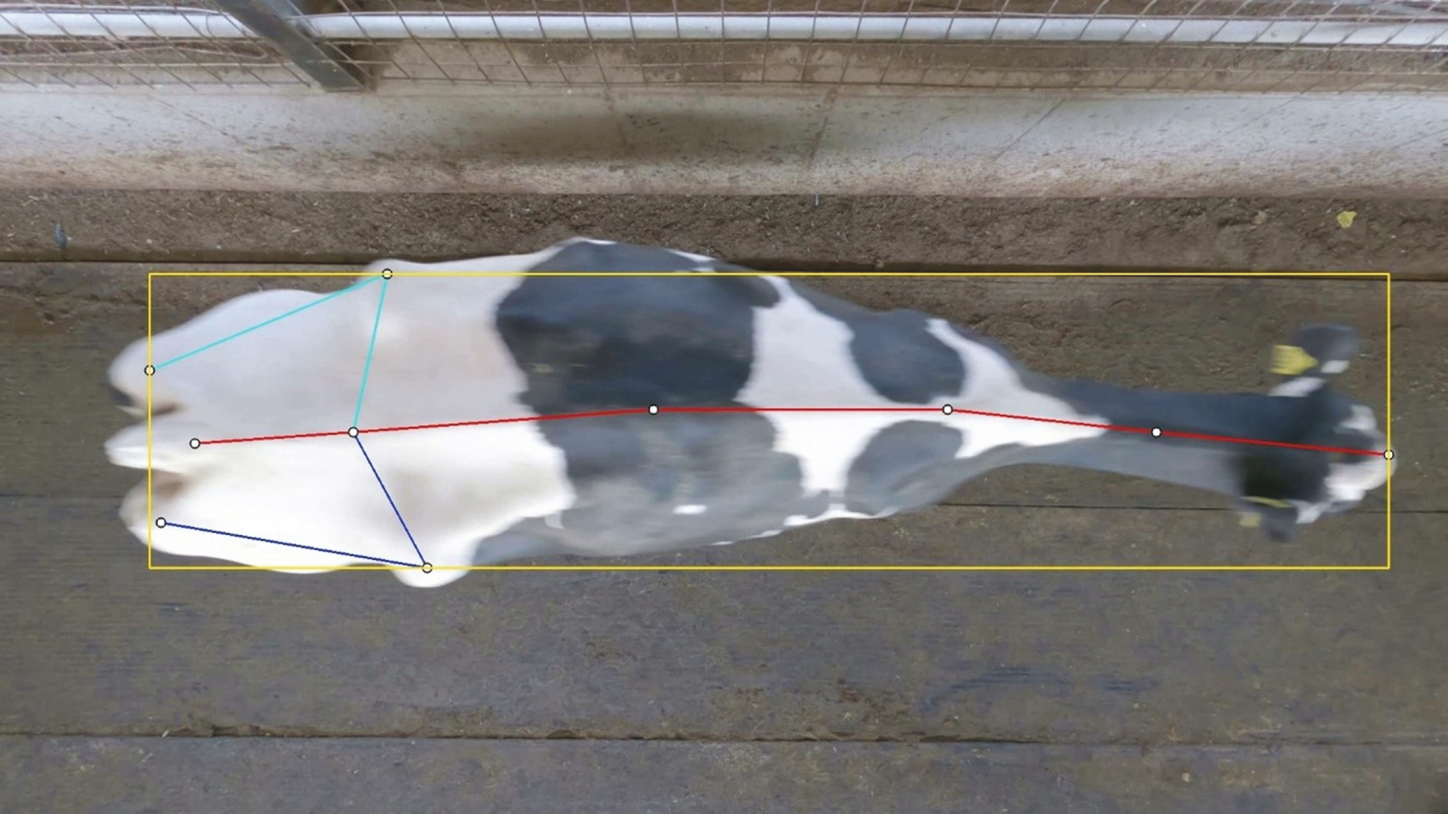
Representation of a top-down view of the image of a dairy cow for lameness detection on commercial settings.

A recent review by Siachos et al. (2024) described that most of the current literature on automated lameness detection is done utilizing CVS. However, a current limitation of CVS in evaluating lameness is the position of the cameras. Generally, studies have cameras placed on alleyways and use a side view of the animal to measure locomotion scores (Jiang et al., 2020; Zheng et al., 2023; Higaki et al., 2024). Detection of lameness utilizing side view recordings is subject to major data loss caused by disturbances such as motion blur, light, and image occlusion caused by other cows, birds, or barn railings (Zheng et al., 2023). Thus, there is an opportunity to utilize alternative camera placing strategies that provide top-down views of the animals to prevent data loss and increase the applicability of CVS for lameness detection in commercial settings (Figure 3).

Anagnostopoulos et al. (2023) validated a commercially available CVS lameness detection system that utilizes top-down images to measure locomotion scores. The authors evaluated the CVS across three commercial dairy farms and reported over 80% agreement between the CVS and human assessments. These results represent a promising application of top-view images for CVS to predict locomotion scores and identify lameness. However, because the authors evaluated a commercially available product, the key features and models utilized by the system to detect lameness are likely proprietary, which prevents it from being further developed by independent parties. Therefore, future research should focus on the development of lameness detection models based on top-down view images and improve the application of CVS on large scales.

Because of the association between mastitis and inflammatory response, the current literature on CVS for mastitis detection relies mostly on the use of infrared thermography (IRT) to detect changes in udder and eye temperature. For example, Xudong et al. (2020) utilized an IRT camera positioned at the entrance of the milking parlor and a deep learning network to detect subclinical mastitis in dairy cows using eye and udder temperature. Their proposed system was able to detect subclinical mastitis with 83.3% accuracy, 92.3% sensitivity, and 76.5% specificity. Similarly, Wang et al. (2022) added an extra IRT camera to the previously described system to record temperature on both sides of the cow’s udder. The authors reported 87.6% accuracy, 96.3% sensitivity, and 84.6% specificity for the detection of subclinical mastitis. Despite the promising results reported, IRT adoption is still limited as the cameras have a limited detection range and can be affected by environmental temperature. An alternative to the use of IRT cameras is to utilize 2D and 3D cameras to monitor changes in udder and teat morphometry (Shorten, 2021) that could be associated with mastitis.

Increased respiration rates have been associated with heat stress incidence in dairy cows and bovine respiratory disease in calves. Respiration rates are normally assessed by visually counting flank movements over a predetermined period of time (i.e., 10, 20, or 30 s) and can range between 26 to 50 breaths/min in cows (Reece et al., 2015) and 24 to 27 breaths/min for calves (Lohr et al., 2016) under normal conditions. However, assessing respiration rates can be labor-intensive and time-consuming as it is based on visual observation. In this context, CVS has been used to measure respiration rates in dairy cattle production systems. For example, IRT has been utilized to assess respiration rates in cows (Stewart et al., 2017) and calves (Lowe et al., 2019). In addition to the IRT limitations previously discussed in this review, these studies did not have an automation factor associated with respiration rate assessment and relied solely on visual assessment of air inhaled and exhaled through an animal’s nostrils. Recently, Shu et al. (2024) proposed a comprehensive pipeline for the noncontact measurement of respiration rates in dairy cows within a free-stall barn using CVS, achieving a Pearson correlation of 0.94 and an RMSEP of 5.35 breaths/min. Mantovani et al. (2023) also utilized CVS to estimate the respiration rate in dairy cows reporting RMSEP and *R*^2^ of 8.3 breaths/min and 0.77, respectively. Furthermore, Mantovani et al. (2023) conducted an external validation of their model using dairy calf videos and reported RMSEP of 13.0 breaths/min and *R*^2^ of 0.73. This opens the possibility for future research to investigate the adoption of CVS to measure respiration rates in calves as a predictor of bovine respiratory disease.

All described studies have shown promising results at the herd level for monitoring animal health. However, as discussed previously, the major challenge in implementing these tools is animal identification. For example, providing the average locomotion score in one barn is important for decision-making, as well as the quality of the stalls and nutrition. However, individual tracking is crucial for interventions at the animal level to improve health and welfare. The same concept applies to respiratory rate; while heat stress generally affects the herd, bovine respiratory disease can affect some individuals and alter their respiratory patterns.

To conclude this section, several phenotypes at the herd level are ready to be implemented under real-world conditions. In this context, future studies should focus on applications at the individual animal level to improve decision-making by leveraging large datasets collected from low-cost sensors and cameras. This is critical for addressing current challenges on farms. For example, in the second-largest milk-producing state in the United States, over 25.6% of farmers face medium or high levels of concern regarding barriers such as data privacy and security issues, keeping up with technological advancements, discomfort with new technologies, costs, data usage, limited training opportunities, poor local infrastructure, a shortage of internet service providers, hardware vendors, and applicable software (Drewry et al., 2019).

## Large Language Models

This section explores the potential applications of LLMs in dairy farming. To facilitate understanding of LLMs’ functionalities, we briefly introduce key concepts in NLP and language models. Next, we explore how LLMs can be used to extract information from data originating from various sources and facilitate dimensionality reduction, data integration, knowledge retrieval, and summarization, specifically in the context of dairy farming.

### Introduction to transformers and language modeling

Transformers are currently the main deep-learning architecture behind LLMs. Proposed by Vaswani et al. (2017), transformers were initially designed for machine translation, but their flexibility and complexity have since enabled their use in other tasks such as language understanding (Dosovitskiy et al., 2020), computer vision (Devlin et al., 2018), question answering (Vaswani et al., 2017), and multimodal representation learning (Radford et al., 2021). The transformer technique is based on the attention mechanism to identify important regions of text and how words are interconnected within sentences. First, the input text is converted to a sequence of vectors called embeddings via a lookup table that maps unique pieces of text, called tokens, to their vector representations (token embeddings), which are learned during training. Next, a multihead attention mechanism assigns various levels of importance to each token within the context of the input text, enhancing the signal of tokens considered important for the training task. In this review, we focus on proposed applications of transformers as they are currently the state-of-the-art architecture for many NLP tasks.

When training language models, the objective is to learn the intrinsic relationships between different words in a language, and in their simplest form, language models are trained to perform next-token prediction (Vaswani et al., 2017). More complex schemes have been proposed for training transformer-based language models, such as masked token prediction and next-sentence prediction (Devlin et al., 2018), sequence-to-sequence denoising (Lewis et al., 2019), and jointly learning textual and visual representations (Radford et al., 2021). The flexibility of transformer-based language models, which essentially perform text-to-text prediction, enables their application in a multitude of downstream tasks and datasets without the need for a specialized architecture. The encoders and decoders trained via self-supervised language modeling schemes can be reused in different tasks such as text classification, text summarization, named entity recognition, question answering, and sentiment analysis with minimal fine-tuning and task-specific training required (Devlin et al., 2018). By training transformer-based language models on a large corpus of text, relationships between words can be extracted in the context of the input text, facilitating fine-tuning for downstream tasks (Vaswani et al., 2017).

### Prompt engineering

Although pretrained models like Bidirectional Encoder Representations from Transformers (BERT) (Devlin et al., 2018) require minimal training to perform different NLP tasks, they still necessitate some degree of fine-tuning. More recently, larger-sized models like GPT-3 (Generative PreTrained Transformer) (Brown et al., 2020), which contains 175 billion parameters (while the original BERT contains at most 340 million), can be prompt-engineered to achieve similar results in various NLP tasks, as demonstrated by (Brown et al., 2020). This represents a breakthrough in AI for NLP, reducing the need for prohibitively expensive computational resources for training such large models from scratch and enabling end users to perform specific tasks using a single pretrained model and prompt engineering.

Prompt engineering consists of designing instructions that can be interpreted by LLMs to perform a customized task. A prompt can be a query, a command, or textual context to be provided to the LLM before performing a certain task. For example, a dataset containing the parity, BW, BCS, and current DIM of individual dairy cows could be provided to the LLM as a form of training data, and a query like “What is the expected daily milk production for a Holstein cow in its second lactation, weighing 1,500 pounds, with BCS 3.75, 70 DIM, eating 21 kg of dry matter (diet composition provided)?” could be provided to perform inference on a new cow. At the time of writing this review, prompt engineering is a rapidly growing research field with numerous applications being proposed every month.

### Embeddings

In the context of NLP, embeddings consist of vector representations of text (tokens, words, sentences, paragraphs, or full articles), which can be processed by subsequent layers of the NLP neural network, which in the case of transformers contain multihead attention layers (Vaswani et al., 2017). At the token level, lookup tables are learned during training to convert text into a latent space representation that distributes tokens based on their semantic and syntactic relationships. For example, the token for “cow” would be converted to a vector representation that is closer to “mammal” than to “helicopter.” Embeddings can also represent multiple tokens at a time by performing a simple weighted average of the word embeddings (Arora et al., 2017) or by introducing a special token used for text classification (Devlin et al., 2018), for example.

Since LLMs are trained on a very large corpus of text, they learn to extract contextual text embeddings efficiently from any input text and convert the high-dimensional and unstructured input text into a fixed-length vector of floating-point values, which can be subsequently used as feature sets for training machine learning models for downstream tasks. This potentially allows data originating from different sources, which follow different formats and standards, to be integrated seamlessly by converting each piece of data to text and extracting text embeddings that can represent all data as a single fixed-length feature vector. Additionally, text embeddings can be used to convert a larger body of text into a vector representation, which can either be decoded into a summary of the original text (text summarization) or compared with a given text query for knowledge retrieval tasks. Finally, when trained in a multimodal setting such as that proposed by (Radford et al., 2021), text embeddings can be related to image embeddings, which allows for zero-shot classification, image generation based on prompts, image description, and other multimodal tasks.

### Applied examples of LLMs for dairy systems

**Figure 4.**
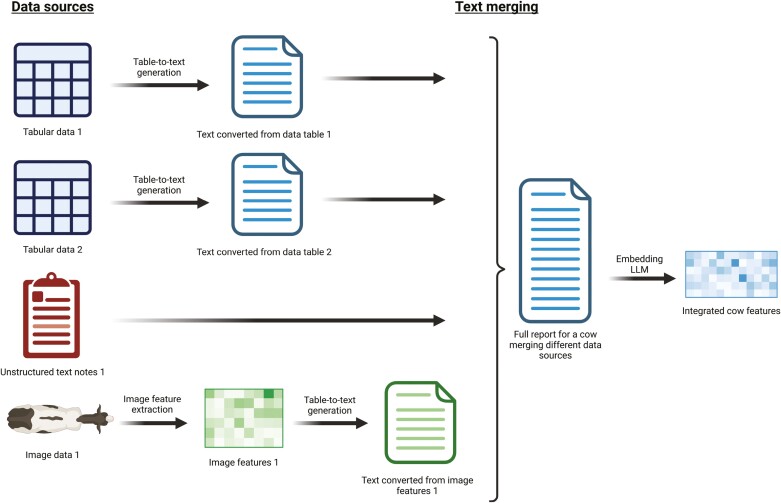
Graphical representation of the utilization of text embeddings and LLMs to integrate data from different sources and formats into standardized features for livestock production.

A current challenge in agriculture that transformers might help address is decreasing data heterogeneity. Using tabular data, traditional data standardization can be done using Python with packages such as Pandas and NumPy, but these tasks often take more than 80% of the analysis time (McKinney, 2012). For this type of task, LLMs such as GPT-3 and BERT can automate the process of standardization. However, the challenge is bigger than anticipated; data standardization in agriculture involves combining data from diverse sources such as wearable sensors, CVS, automatic feeders, milking and feeding systems, and farm management records, all of which have different formats, label quality, and consistency (Cabrera et al., 2020; van der Voort et al., 2021). In this context, LLMs can extract features from each type of data, called modality, and standardize it for future modeling (Lahat et al., 2015), offering the benefit of a lower dimensional space (Huang et al., 2020; Al-Hammuri et al., 2023). In other domains, such as autonomous driving (Cui et al., 2024a) and health care (Cui et al., 2024b), multimodal LLMs have achieved great success in integrating heterogeneous datasets for a multitude of tasks. However, the potential of using LLMs for heterogeneous data integration in agriculture and livestock is yet to be explored. A graphical representation of data integration utilizing text embeddings in dairy cattle production systems is displayed in Figure 4.

Another promising application of LLMs in agriculture and livestock is to improve the accessibility of agricultural research. LLMs enable the extraction of information from vast databases of scientific literature and technical documents to make accurate recommendations. Zhu et al. (2023) proposed using deep learning models to extract knowledge from 20 manuscripts as entities and relations from domain literature related to transition cows. During the training process, the authors applied a Long Short-Term Memory (LSTM) model, a BERT, and transfer learning before fine-tuning the BERT model. They achieved F1 scores of 80%, 23%, and 89%, respectively. The lower result using the BERT model without fine-tuning was likely due to a lack of training data. After model training, users proposed applied questions (i.e., “What diseases are associated with ketosis?”; “What disease would monensin affect?”). The quality of the answers proposed by the model was evaluated by human experts and achieved a 7.5 out of 10 score. Such applications are powerful and can help inform decision-making for farmers, animal scientists, veterinarians, and researchers. In the context of LLMs, retrieval-augmented generation (RAG) can be used for NLP tasks that require specific knowledge that might not have been fully represented in the training text corpus (Lewis et al., 2020). Using this technique, a dataset of scientific papers collected from journals covering topics related to animal science could be used as a reference dataset for knowledge retrieval, and specific questions could be posed to the LLM by extracting embeddings from the query text and comparing them with the text embeddings contained in the scientific reference dataset. This technique allows the LLM to provide answers to the questions using text passages found in the reference dataset, potentially enhancing the transparency and scientific accuracy of such answers.

## Conclusions

There are several current and possible applications for AI tools in dairy systems management. This review highlights the rapidly emerging research field of developing CVS for dairy farming. Furthermore, we observed that the current CVS research is focusing on critical limitations such as the identification of solid-colored animals, open-set settings, semisupervision for image labeling, among others. We highlight the fact that CVS has the potential to phenotype multiple animals using a single sensor in a noninvasive way. Yet, there are still challenges in CVS that need to be addressed, such as data integration, the need for computational infrastructure deployed in farm settings, and connectivity.

Currently, there exists a knowledge gap regarding the use of LLMs in agricultural and livestock systems, highlighting the potential for future research to explore their capabilities. Despite the likely need for adapting LLMs for their adequate use in agriculture and livestock farming, such as by fine-tuning the models or performing RAG using agricultural research articles, LLMs have the potential to minimize major challenges in the dairy industry related to data heterogeneity and data integration via text embedding extraction. Furthermore, LLMs can be further utilized to increase the accessibility of information to farmers through knowledge retrieval and text summarization techniques, democratizing the access to useful information for making on-farm management decisions.

## About the Authors

 **Guilherme L. Menezes** is a postdoctoral research associate in the Department of Animal and Dairy Science at the University of Wisconsin–Madison. He earned his master’s and PhD in Animal Sciences from the Federal University of Minas Gerais, Brazil. He has developed computer vision systems for evaluating critical traits in cattle and predictive analytics for early detection of health issues and animal performance. Currently, his research combines digital technologies such as drones and satellite imagery to assess pasture quality and animal behavior, with a focus on improving pasture management and sustainability in grazing systems.

 **Gustavo Mazon** is a postdoctoral research associate in the Department of Animal and Dairy Sciences at the University of Wisconsin–Madison. He earned his master’s and PhD from the University of Kentucky, specializing in precision technologies and nutritional interventions for dairy cattle. Currently, he assesses technology adoption by dairy farmers and develops educational resources to promote artificial intelligence and data technology in livestock production.

 **Rafael E.P. Ferreira** is an Electrical and Computer Engineer and postdoctoral research associate in the Department of Animal and Dairy Sciences at the University of Wisconsin–Madison. He earned his master’s in computer science and PhD in dairy science at the University of Wisconsin–Madison, specializing in developing machine learning and computer vision systems to improve dairy farm management and phenotyping. Current research is focused on the early detection of subclinical ketosis in dairy cows during the transition period by integrating data from multiple sensors and exploring computer vision and natural language processing techniques.

 **Victor E. Cabrera** is a professor at the University of Wisconsin–Madison, where he combines his expertise in dairy science with applied economics and cutting-edge technology. A pioneer in the application of AI to dairy management, Dr. Cabrera’s work, particularly on the Dairy Brain project, has significantly influenced global dairy farming practices. Dr. Cabrera has authored numerous influential publications that effectively bridge the gap between complex data analytics and practical applications, making substantial contributions to the field.

 **Joao R.R. Dorea** is an Associate Professor at the University of Wisconsin–Madison, affiliated with the Department of Biological Systems Engineering and the Data Science Institute. His research focuses on leveraging artificial intelligence and machine learning to build predictive models for optimal farm management decisions. His team is dedicated to enhancing animal health and welfare, as well as improving the production efficiency of livestock systems through these digital technologies. **Corresponding author**: joao.dorea@wisc.edu
